# The Role of Multidisciplinary Approaches in the Treatment of Patients with Heart Failure and Coagulopathy of COVID-19

**DOI:** 10.3390/jcdd10060245

**Published:** 2023-06-03

**Authors:** Katarzyna Gryglewska-Wawrzak, Krzysztof Cienkowski, Alicja Cienkowska, Maciej Banach, Agata Bielecka-Dabrowa

**Affiliations:** 1 Department of Cardiology and Congenital Diseases of Adults, Polish Mother’s Memorial Hospital Research Institute (PMMHRI), 93338 Lodz, Poland; maciejbanach77@gmail.com (M.B.); agatbiel7@poczta.onet.pl (A.B.-D.); 2 Faculty of Medicine, Medical University of Lodz, 90419 Lodz, Poland; krzysztof.cienkowski@stud.umed.lodz.pl; 3 Faculty of Biology and Environmental Protection, University of Lodz, 90136 Lodz, Poland; a.cienkowska7@gmail.com; 4 Department of Preventive Cardiology and Lipidology, Medical University of Lodz, 90419 Lodz, Poland

**Keywords:** COVID-19, heart failure, inflammation, cardiopulmonary exercise testing, coagulopathy

## Abstract

Coronavirus disease 2019 (COVID-19) is a severe respiratory syndrome caused by severe acute respiratory syndrome coronavirus 2 (SARS-CoV-2). Heart failure (HF) is associated with a worse prognosis for patients with this viral infection, highlighting the importance of early detection and effective treatment strategies. HF can also be a consequence of COVID-19-related myocardial damage. To optimise the treatment of these patients, one needs to understand the interactions between this disease and viruses. Until now, the validity of the screening for cardiovascular complications after COVID-19 has not been confirmed. There were also no patients in whom such diagnostics seemed appropriate. Until appropriate recommendations are made, diagnosis procedures must be individualised based on the course of the acute phase and clinical symptoms reported or submitted after COVID-19. Clinical phenomena are the criteria for determining the recommended test panel. We present a structured approach to COVID-19 patients with heart involvement.

## 1. Introduction

Coronavirus disease 2019 (COVID-19) is an infectious disease caused by coronavirus 2 of severe acute respiratory syndrome (SARS-CoV-2). It started as an epidemic on 17 November 2019 in Wuhan, Hubei Province, central China, and was declared a pandemic by the World Health Organisation (WHO) on 11 March 2020. At the end of 2022, more than 670 million cases of the SARS-CoV-2 virus has been registered in 192 countries and territories. There are nearly 20 million active cases, more than 640 million recoveries, and more than 6.60 million deaths. Typical symptoms of the disease include fever, dry cough, anosmia (loss of smell), ageusia (loss of taste), fatigue, and dyspnoea. Less common symptoms include sputum production, headache, chills, haemoptysis, chest pain, diarrhoea, nausea and vomiting, and a sore throat [1]. Most cases of the disease are mild, but some may lead to pneumonia or multiple organ failure. Some patients have only gastrointestinal symptoms. Developing a viral infection can lead to pneumonia, acute respiratory distress syndrome (ARDS), sepsis and septic shock, and death. It can also cause a number of cardiac complications, such as arrhythmias, cardiogenic shock, acute myocardial injury, and ST changes on the electrocardiogram (ECG) [2]. In a multicentre cohort study, the authors found a high incidence of heart failure (23%) as an extrapulmonary manifestation of infection, and the rate was higher (52%) in non-survivors [3]. It is still debatable whether this is due to viral myocardial disease or as a result of the cytokine storm and rapid inflammatory response. The pathophysiology of SARS-CoV-2 is characterised by the overproduction of inflammatory cytokines leading to systemic inflammation and multiorgan dysfunction syndrome, which acutely affects the cardiovascular system.

**Figure 1:**
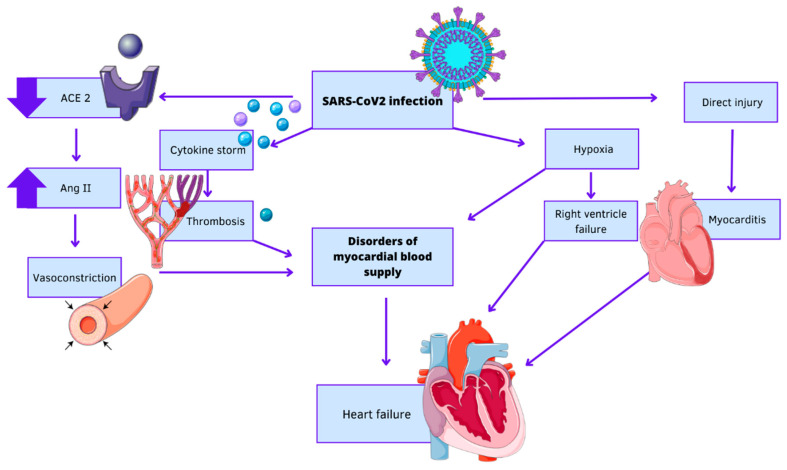
Mechanism of development COVID-19-induced heart failure. The figure was partly generated using Servier Medical Art, provided by Servier, licenced under a Creative Commons Attribution 3.0 unported licence.

The mechanisms of cardiovascular injury caused by SARS-CoV-2 infection are not fully understood, but the primary mechanism involves the virus infiltrating host cells via angiotensin converting enzyme 2 (ACE2). ACE2, a membrane protein, plays a vital role in mitigating the adverse effects of the renin–angiotensin–aldosterone system (RAAS) by converting angiotensin II (Ang II) to Ang- (1–7) [4]. ACE2 is predominantly present in the vascular endothelium of various tissue structures, including lung cells, smooth muscle cells within the pulmonary vascular system, bronchial epithelium, epithelial cells in the lungs, heart, blood vessels, intestine, kidneys and testis [5]. As a result of SARS-CoV-2 infection, virus attachment to ACE2 receptors located on the surface of cardiac epithelial cells leads to the direct harm to the myocardium and subsequent dysfunction, potentially contributing to the overall damage—Figure 1. The significant presence of ACE2 in cardiomyocytes, fibroblasts, endothelial cells, epicardial adipocytes, and smooth muscle cells further reinforces the theory of viral injury occurring directly [6]. The presence of cardiovascular complications in individuals with COVID-19 leads to a more unfavourable prognosis, underscoring the significance of early detection and the implementation of effective therapeutic approaches. Patients who already have underlying conditions like cardiovascular disease face a particularly elevated risk of illness and death resulting from this viral infection. Given the compelling evidence suggesting that myocardial damage worsens the severity of COVID-19, healthcare professionals should prioritize cardiac management. Moreover, multiple studies have shown that COVID-19 can worsen existing cardiovascular conditions and induce new cardiovascular injuries [7,8]. Furthermore, the long-term consequences of COVID-19 on cardiovascular health remain a major global concern.

## 2. COVID-19 and Heart Failure

Heart failure (HF) remains a major clinical and public health concern. This condition is also known as the cardiovascular epidemic of the 21st century [9]. This finding has been reinforced by the observation of an exponential increase in hospitalisations for HF. Current estimates indicate that HF affects more than 64 million people around the world [10]. In 2021, the world’s largest scientific organisations proposed a consensus on the universal definition and classification of HF [11]. HF was defined as a clinical syndrome with symptoms due to a structural and/or functional abnormality of the heart, confirmed by elevated levels of natriuretic peptides and/or objective evidence of pulmonary or systemic congestion. HF was divided into three categories according to left ventricular ejection fraction (EF): HF with reduced EF (HFrEF), slightly reduced (HFmrEF), and preserved EF (HFpEF), according to the ranges of EF <40%, 41–49%, and 50%, respectively. Furthermore, a new unit was introduced, i.e., HF with EF improvement, which was defined as HF with baseline EF <40% with a 10-point increase in EF from baseline value and a second measurement of EF > 40% [12]. Trends in HF show an overall increase in HF prevalence; however, when data are analysed according to EF, the prevalence of HFpEF has been observed to increase, but it is stable or even decreases for HFrEF [13]. Understanding the pathophysiological mechanism leading to heart failure is crucial for the selection of appropriate therapeutic options. After myocardial damage (e.g., myocardial infarction, infection), cellular and neurohumoral processes occur, the consequence of which is the activation of the sympathoadrenergic system and renin–angiotensin–aldosterone. This phenomenon leads to adaptive mechanisms accompanied by volume overload, tachycardia, shortness of breath, and further deterioration of cellular function [14].

Inflammation is an important factor in the development of HF. Previous research has shown that inflammation contributes to the pathogenesis of HFrEF [15,16,17]. However, the contradictory results of several trials of anti-inflammatory drugs led to the conclusion that inflammation played a role in the development of HFrEF, but it is more likely not to be a primary cause [18]. In contrast, inflammation is believed to promote the development of HFpEF [19]. Several studies have shown that increased levels of inflammatory molecules such as C-reactive protein (CRP), tumour necrosis factor-alpha (TNFα), interleukin-1 (IL-1), growth differentiation factor 15 (GDF15), soluble ST2, and pentraxin-3 are more noticeable in HFpEF than in HFrEF [20,21,22,23]. In the work of Chuda-Wietczak, the authors showed that elevated CRP (>2.38 µg/mL, OR = 2.93, 95% CI = 1.31–6.54, *p* = 0.007) was an independent prognostic factor for adverse clinical events (CE) in the population with HF [24]. Myocarditis is often considered a potential cause of HF in patients with COVID-19. However, some reports have indicated that myocarditis is not commonly observed in severe cases of COVID-19 and it tends to be mild [25,26].

The relationship between COVID-19 and heart failure is complex. Pro-inflammatory cytokines triggered by SARS-CoV-2 may indirectly lead to cardiac damage. Several clinical studies on COVID-19 patients reported significantly elevated inflammatory biomarkers in circulation, including interleukin (IL)-2, IL-6, IL-7, monocyte chemoattractant protein 1 (MCP-1), macrophage inflammatory protein 1-α (MIP-1α), tumour necrosis factor-α (TNF-α), interferon-γ inducible protein (IP)-10, CRP, ferritin, and procalcitonin [7,27]. Although triggered by local infection in the lungs, increased systemic levels of these inflammatory cytokines activate inflammatory and maladaptive remodelling pathways in multiple organs, including the heart.

The COVID-19 outbreak has an impact on HF management, with a decrease in hospitalisation for HF during the outbreak, leading to an increase in HF mortality. HF can be a consequence of COVID-19-related myocardial damage. HF history is a risk factor for more serious clinical cases of COVID-19. Patients with HF are more likely to develop a myocardial injury. The history of HF has also been found to be associated with an increased risk of hospitalisation and a severe clinical course in patients with COVID-19. Observation studies in patients hospitalised with COVID-19 detected vascular damage based on troponin levels and defined it as an increase greater than the normal 99 percentile. Blood pressure levels increased by 8–12% in unselected cases of COVID-19, increased from 23% to 33% in critically ill patients, and increased further when considered in patients with heart disease [28,29,30]. Some studies have also evaluated plasma concentrations of N-terminal-pro-brain natriuretic peptides (NT-proBNP) and have shown that these concentrations are higher in patients with myocardial injuries, although these are not independently associated with the results [30,31]. Natriuretic peptides have been found to be elevated in patients with COVID-19, even in the absence of heart failure. Due to this factor, the diagnosis of HF can be challenging in this group of patients. In the study of Bergami et al., the authors included COVID-19 patients and demonstrated that 53.4% had elevated BNP levels. High BNP levels were also strongly associated with an increased risk of AHF (OR 19.9; 95% CI 8.6–45.9; *p* < 0.001), a correlation that persisted both in patients with and without a prior cardiovascular disease history (*p* for interaction = 0.29). Subjects with increased BNP also had a higher likelihood of developing ARF (OR 2.7; 95% CI 2.1–3.6; *p*-value < 0.001), even in the absence of AHF (OR 3.00; 95% CI 2.20–4.1; *p*-value < 0.001) [32]. This finding supports the recommendation to regularly utilize BNP testing for all COVID-19 patients admitted to the hospital, regardless of their previous cardiovascular disease history.

Cardiovascular complications, including HF, have been associated with other viral respiratory diseases. Infection with respiratory syncytial virus (RSV) infection frequently results in the onset of HF due to pre-existing cardiopulmonary and immunoprophylaxis conditions [33]. The influenza virus is known to have significant impacts on inflammatory processes and is a common trigger for cardiovascular diseases [34]. The potential relationship between influenza infection and the emergence of HF involves various pathophysiological disruptions. These include hypoxemia, activation of neuroendocrine and sympathetic systems, volume overload due to cardio-renal injury, direct injury to cardiomyocytes, and concurrent hyperinflammation. These factors interact with each other, contributing to the progression of HF [35]. According to a study conducted by Kytomaa et al., the analysis of community surveillance data was utilized to examine the occurrence of myocardial infarction (MI) and hospitalizations for heart failure in relation to monthly influenza activity. The findings demonstrated that a 5% monthly rise in influenza activity was linked to a 24% increase in hospitalizations for heart failure, with an incidence rate ratio (IRR) of 1.24 (95% CI, 1.11–1.38; *p* < 0.001) [36]. In contrast to the COVID-19 pandemic, a decrease of 50% in heart failure hospitalizations has been observed since the first diagnosed case of COVID-19 [37]. A similar downward trend has been observed in acute cardiovascular hospitalizations, with a significant decrease in the daily hospitalization rate throughout March 2020 (at a rate of −5.9% per day, ranging from −7.6% to −4.3%, *p* < 0.001) [38]. These reductions occurred despite a significant increase in mortality (up to 90%) attributed to cardiovascular diseases during this period, along with a temporary doubling of out-of-hospital cardiac arrest incidences [39]. This suggests that the decrease in hospitalizations can largely be attributed to patients’ fear of seeking healthcare in medical facilities due to concerns about contracting the virus. This fear is further supported by the widespread adoption of physical distancing and isolation measures.

The clinical manifestations of the myocardial injury caused by SARS-CoV-2 include arrhythmia and sudden cardiac death, pulmonary embolism, acute coronary syndromes, myocarditis, acute heart failure, and cardiogenic shock [40]. Furthermore, it should be noted that distant cardiovascular complications can also occur in patients with mild or non-symptomatic courses of COVID-19. 

Several studies have shown the adverse effects of COVID-19 infection on pre-existing cardiovascular disease. In a study by Inciardi et al., the authors compared the clinical presentations and outcomes of patients (*n* = 99) with and without cardiac disease hospitalised for COVID-19. Among cardiac patients, 40% had a history of heart failure, 36% had atrial fibrillation, and 30% had coronary artery disease. Mortality was higher in patients with cardiac disease compared to the others [41]. Patients may have experienced acute heart failure (AHF) in addition to chest pain, suggesting myocardial ischemia or myocardial ischemia and palpitations. After ARDS and sepsis, HF was the leading cause of death in 113 deaths from COVID-19 [42]. Subsequent studies have also shown a worse prognosis in patients with COVID-19 who had known cardiovascular disease [43,44,45,46]. In the study of Bhatt et al., data from 1,212,153 HF patients were analysed. The authors revealed that hospitalisation with COVID-19 was associated with greater odds of in-hospital mortality as compared to hospitalisation with acute HF. In total, 24.2% of patients hospitalised with COVID-19 died in hospital compared to 2.6% of those hospitalised with acute HF [47]. Sokolski et al. compared the outcomes of hospitalised COVID-19 patients with HF and patients with other cardiovascular diseases. This study demonstrated an increased risk of in-hospital death in patients with HF [48]. Other research studies confirmed a higher risk of death in patients with COVID-19 [49,50,51,52].

Patients who have been infected with SARS-CoV-2 may also be at risk of developing heart failure. Rey et al. collected data from 3080 patients with confirmed infection and 30 days after infection. Patients with COVID-19 had a significant incidence of AHF, which was associated with very high mortality rates. Furthermore, patients with a history of chronic heart failure (CHF) were prone to developing acute decompensation after a diagnosis of COVID-19 [53]. The study of Zaccone et al. has shown that COVID-19 could be an independent risk factor for the development of HFpEF [54]. In another meta-analysis, the authors demonstrated acute HF as a frequent complication of COVID-19 infection, associated with a higher risk of mortality in the short term [55].

In our study, we enrolled patients who recovered from COVID-19 three to six months after their confirmed diagnosis. These patients were divided into two groups: study group presented with worse oxygen uptake (VO2) [%VO2pred <80%; *n* = 47 at a median age of 49 years, median VO2max 17 mL/kg/min]; and a control group with a median age 55 years who had VO2pred <80% [73 patients, median 23 mL/kg/min]. There was a higher proportion of men and a greater percentage of total body water content (TBW%) in the study group compared to the control group (53% vs. 29%, *p* = 0.007, and 52.67% (±6.41) vs. 49.89% (±4.59), *p* = 0.02, respectively). The group with %VO2pred <80% presented with significantly lower late diastolic fill velocity (A), global peak systolic strain (GLPS), and annular tricuspid plane systolic excursion (TAPSE) compared to the control group [median 59.5 (IQR: 50.0–71.0) vs. 70.5 (IQR: 62.0–80.0) cm/s, *p* = 0.004; 19.34 (±1.72) vs. 20.10 (±1.35) %, *p* = 0.03; 21.86 (±4.53) vs. 24.08 (±3.20) mm, *p* = 0.002; respectively]. Multiple logistic regression analysis indicated that velocity (A) and male gender were independently associated with %VO2pred [OR 0.40, 95% CI 0.17–0.95, *p* = 0.03; OR 2.52, 95% CI 1.07–5.91, *p* = 0.03; respectively]. Lower velocity (A), TAPSE, GLPS and hydration status are related to limited exercise tolerance after COVID-19 in patients with normal left ventricular ejection fraction [56]. Based on these results, patients with long-COVID without heart failure diagnosis may have worse echocardiographic parameters of diastolic dysfunction. Ergospirometry can be useful in assessing the risk of developing heart failure in this group of patients. Further analyses are warranted. Other studies have also shown echocardiographic abnormalities in survivors of COVID-19 [57,58].

Currently, the validity of cardiovascular screening after COVID-19 has not been confirmed, and there are no established guidelines for such diagnostics. However, some studies suggest that the screening may be useful in patients with post-acute COVID-19 syndrome [59,60]. Until appropriate recommendations are made, diagnosis procedures must be individualised based on the course of the acute phase and clinical symptoms reported or submitted after COVID-19. Clinical phenomena are the criteria for determining the recommended test panel. The most common diseases reported to patients after infection include exercise intolerance, which usually requires a different diagnosis (cardiac disease, pulmonary disease, muscle loss, or mental illness) [57]. Table 1 summarises the results of the studies mentioned above.

## 3. Diagnosis of Heart Failure in Patients after COVID-19

### 3.1. Clinical Examination

Thorough subjective and physical examinations allow one to raise suspicion of the disease and plan further diagnostic procedures. The subjective examination is the first examination that, when properly conducted, allows one to propose a correct initial diagnosis of the disease in many patients. For patients who present symptoms of heart failure for the first time to a primary care physician or cardiology clinic, the physician first assesses the likelihood of a disease based on the history of coronary artery disease, high blood pressure, and other diseases and conditions that can cause heart failure. The doctor then examines the patient for signs of heart failure [61,62].

### 3.2. Laboratory Tests

The following parameters are of particular importance in the diagnosis of heart failure [12]:

1. concentration of natriuretic peptides in plasma—to exclude HF: in a patient without acute worsening of symptoms, HF is unlikely when BNP < 35 pg/mL (<105 pg/mL in atrial fibrillation), NT-proBNP < 125 pg/mL (<365 pg/mL in atrial fibrillation);
2. arterial blood gas analysis for detection of respiratory failure;
3. serum troponin for detection of acute coronary syndrome (ACS);
4. blood urea nitrogen, serum creatinine, electrolytes—for the detection of renal dysfunction;
5. full blood count—anemia may exacerbate or cause CHF;
6. transferrin, ferritin, signs of iron deficiency, most often of a functional nature—reduced transferrin iron saturation; a decrease in ferritin usually occurs only with absolute iron deficiency (it may not occur in the presence of inflammation);
7. inflammatory cytokines (C-reactive protein, procalcitonin)—for the diagnosis of infection;
8. increased activity of aminotransferases and lactate dehydrogenase (LDH), increased concentrations of bilirubin in plasma—in patients with venous stasis in the systemic circulation, with hepatomegaly;
9. the concentration of thyroid stimulating hormone (TSH), because thyroid disease can mimic or worsen the symptoms of HF;
10. D-dimer—when pulmonary embolism (PE) is suspected.

There are established guidelines for managing outpatients with suspicion of PE [63], based on clinical probability assessment and D-dimer dosage. Outcome studies have shown that the 3-month thromboembolic risk is <1% in patients with low or intermediate clinical probability and D-dimer < 500 ng·mL^−1^ who are left untreated [64]. The adjust-PE study has demonstrated that the D-dimer level adjusted to patient age, with higher thresholds in older patients (age × 10 ng·mL^−1^), can safely rule out PE [65]. Hypercoagulability and the need to prioritise coagulation markers for prognostic abilities have been highlighted in COVID-19. In a meta-analysis, the authors included 113 studies (*n* = 38,310) and showed that higher D-dimer levels provide prognostic information useful for clinicians to assess early COVID-19 patients at risk for disease progression and mortality outcomes [66].

### 3.3. Electrocardiogram (ECG)

The ECG usually reveals features of the underlying disease—ischemic heart disease, arrhythmias or conduction disorders, hypertrophy, or overload [67].

### 3.4. Chest Radiograph

The chest radiograph generally reveals enlargement of the heart (except in most cases of hyperkinetic states and diastolic insufficiency), signs of venous congestion in the pulmonary circulation [68].

### 3.5. Echocardiography

1. Left ventricular systolic function—by analysing segmental and global left ventricular contractility and left ventricular ejection fraction (LVEF) measurement (Simpson method; <40% indicates significant left ventricular systolic dysfunction; values 41–49% are considered the so-called grey zone and one of the diagnostic criteria HFmrEF—a complete differential diagnosis of noncardiac causes of symptoms is necessary, as in HFpEF) [12].
2. Left ventricular diastolic function—transmitral E/A ratio and E velocity deceleration time (DT), e’ velocity (average and absolute value of septal and lateral side) of the mitral annulus by pulsed tissue Doppler, E/e’ ratio, and the estimate of systolic pulmonary artery pressure (sPAP) derived from tricuspid regurgitation (TR) velocity [69].
3. Anatomical abnormalities, hypertrophy, dilation of the heart chambers, valvular defects, congenital defects. Additional evaluation of many parameters of cardiac structure and function is of particular importance in differential diagnosis, especially with LVEF <40%. In some cases (e.g., poor imaging conditions on transthoracic examination, suspected prosthetic valve dysfunction, detection of a thrombus in the left ear in patients with atrial fibrillation, diagnosis of bacterial endocarditis or congenital defects), transoesophageal echocardiography is indicated [70].
4. Signs of PE—dilation of the right ventricle (RV), pulmonary ejection acceleration time <60 ms with a peak systolic tricuspid valve gradient < 60 mmHg [63]. Echocardiographic examination is not mandatory as part of the routine diagnostic workup in haemodynamically stable patients with suspected PE. In case of suspected high-risk PE, the absence of echocardiographic signs of RV overload or dysfunction practically excludes PE as the cause of hemodynamic instability [71].

### 3.6. Computed Tomographic Pulmonary Angiography (CTPA)

CTPA is the first-line imaging technique in patients with suspected PE and is indicated as a class IC procedure for individuals with a high suspicion of PE, even in cases of hemodynamic instability. In patients with low or moderate clinical probability, a correct CTPA result can be sufficient to rule out the diagnosis of PE without the need for additional testing (class IA) [63].

### 3.7. Compression Ultrasonography (CUS)

CUS is a relevant tool for diagnosing deep vein thrombosis (DVP). This condition is a major medical problem that accounts for most cases of pulmonary embolism [72].

### 3.8. Cardiopulmonary Exercise Testing (CPET)

CPET is a relevant tool in patients with long-COVID. This examination is helpful in the case of a discrepancy between the severity of symptoms and the objective parameters of the severity of the disease, and when distinguishing between cardiac and pulmonary causes of dyspnea [73]. In one meta-analysis, the authors demonstrated that exercise capacity was reduced more than 3 months after SARS-CoV-2 infection among individuals with long-COVID symptoms compared with individuals without symptoms [74].

**Figure 2:**
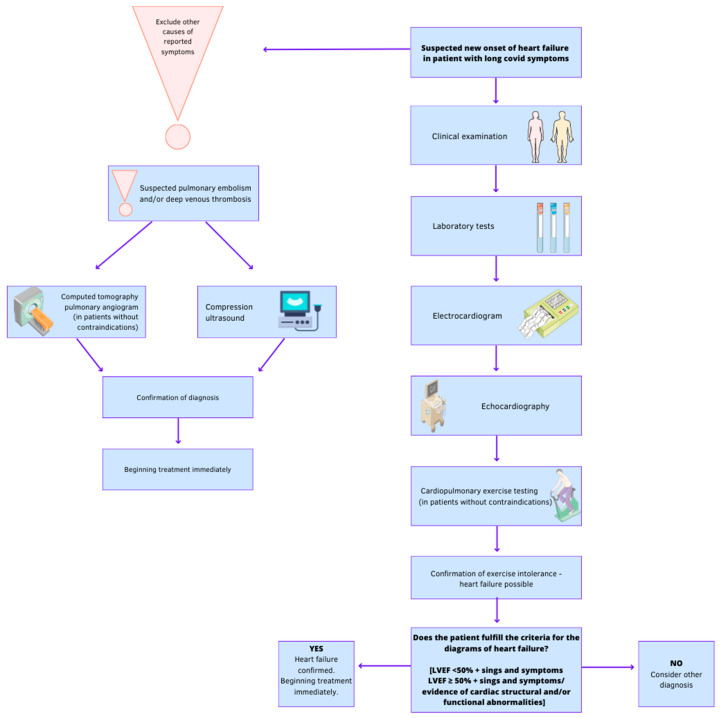
Management in long-COVID patients with suspected heart failure. The figure was partly generated using Servier Medical Art, provided by Servier, licenced under a Creative Commons Attribution 3.0 unported licence; one of the icons was from Flaticom.com (accessed on 18 April 2023).

Figure 2 demonstrates proposed management in long-COVID patients with suspected heart failure. 

## 4. Treatment of Heart Failure after COVID-19

The management of HF in patients with or after COVID-19 should be performed according to established guidelines and protocols. The pharmacotherapy management for heart failure depends on the haemodynamic status. During the pandemic, there were hypotheses that angiotensin-converting enzyme inhibitors (ACEi), angiotensin receptor blockers (ARB), or angiotensin receptor neprilysin inhibitors (ARNI) may affect mortality in patients with COVID-19, theoretically due to interaction with the bradykinin pathway. Several scientific societies recommend that treatment with RAAS inhibitors (ACEi/ARB/ARNI) should not be discontinued in patients diagnosed with COVID-19 [75]. Interruption is associated with an increased mortality risk [76,77,78]. Safety and efficacy of using sodium-glucose co-transporter 2 inhibitors (SGLT2i) in case of COVID-19 is still debatable. Zhu et al. conducted a meta-analysis in patients with diabetes mellitus and showed that the use of SGLT2i before COVID-19 infection is associated with lower adverse outcomes [79]. More research is needed in patients with HF. Beta-receptor blockers should be used with caution with patients treated with antiviral agents due to the risk of hypotension and bradycardia. Currently, there is no evidence that the four beta-blockers approved for HF treatment (metoprolol, bisoprolol, carvedilol, or nebivolol) are preferred, but experimental studies have shown that carvedilol may offer unique anti-cytokine properties [80]. Currently, there is contradictory evidence regarding the role of mineralocorticoid receptor antagonists (MRA). In a research study, the authors demonstrated that, in patients with COVID-19, MRA treatment had an overall positive impact on all-cause mortality and clinical improvement, most probably through a direct anti-inflammatory effect [81]. In another study, SARS-CoV-2-induced endothelial injury was abrogated by the spironolactone [82]. In a systematic review, an association between MRA therapy and mortality in patients infected with SARS-CoV-2 was explored [83].

## 5. COVID-19 and Coagulopathy

COVID-19 is primarily considered a respiratory infection, but there is increasing evidence of multi-organ complications of the disease. Compared to other common respiratory viral infections, patients with COVID-19 had a higher incidence and severity of blood clotting, usually associated with higher levels of D dimer, C-reactive protein, P-selectin and fibrinogen. The hypercoagulation state of COVID-19 infection is associated with severe inflammatory reactions, cytokine storm, endothelial damage, and clinical complications [84,85]. The mechanisms of coagulopathy are complex in COVID-19. It may involve the common pathways documented in other viral diseases and hospitalized patients, such as endothelium cell damage by viral antigens, upregulation of Toll-like receptors and von Willebrand factors, causing an uncontrolled coagulation similar to disseminated intravascular coagulopathy (DIC) with excess fibrin clot formation. When these fibrin clots are degraded by the body’s own anticoagulant system, fibrin degradation products are produced, including D-Dimer, which is commonly tested in laboratories [86].

Furthermore, the pro-inflammatory state promotes further thrombogenesis by inhibiting anticoagulation processes, such as thrombomodulin, endothelial cell protein C, and the TF pathway inhibitor. Eventually, fibrinolysis is inhibited by the release of plasminogen activator inhibitor-1 (PAI-1) from endothelial cells during inflammation [87].

Due to the unique complications described above, the prolonged anticoagulation in patients with thrombosis during COVID-19 infection should be considered. Lachant et al. demonstrated that chronic anticoagulation at the time of infection may protect against thrombotic complications and decrease disease severity [88]. In one multicentre, randomized trial, patients hospitalised with COVID-19 at increased risk for VTE were randomly assigned (1:1) to receive, at hospital discharge, rivaroxaban 10 mg/day or no anticoagulation for 35 days. In individuals with a high risk of complications who were discharged from the hospital following COVID-19, administering rivaroxaban at a dose of 10 mg per day for a duration of 35 days improved clinical outcomes compared with no extended thromboprophylaxis [89].

Won et al. conducted a study in which the autopsy lungs of COVID-19 patients exhibited severe coagulation abnormalities, immune cell infiltration, and platelet activation [90]. In one small study, the authors examined the lungs of patients who died from COVID-19 and compared them with seven lungs obtained during autopsy from patients who died from ARDS secondary to influenza A(H1N1) infection and uninfected control lungs. Autopsy of the COVID-19 has demonstrated extensive endothelial injury, vascular thrombosis with microangiopathy, occlusion of the alveolar capillaries, and signs of neo-angiogenesis. Additionally, capillary microthrombosis was nine times more frequent in COVID-19 compared to influenza [91]. In a series of autopsy of COVID-19 patients in Germany, 58% of patients with blood thromboembolism did not suspect thromboembolism before death [92].

Many studies have focused on the risk factors for the development of coagulopathy in COVID-19 and determining its relationship with the severity of the infection. A meta-analysis showed an association between cardiac injury in COVID-19 and developing coagulopathy [93]. Jin et al. demonstrated that coagulation dysfunction was frequent in Chinese COVID-19 patients. Non-survivors had significantly higher levels of D-dimer, prolonged prothrombin time (PT), and decreased platelet counts compared to survivors [94]. Similar results were also obtained in another research [95]. The study of Zhu demonstrated a high prevalence of coagulopathy in patients with severe COVID-19 [96]. Other meta-analyses confirmed the association between coagulopathy and a poor prognosis of SARS-CoV-2 infection [97,98]. In the Agarwal meta-analysis, 28 studies included 6053 patients with SARS-CoV-2 infection. The authors demonstrated that venous thromboembolic events in COVID-19 were associated with male gender [99]. Another study showed that immune thrombocytopenia (ITP) secondary to SARS-CoV-2 infection was more prevalent in men (54.8%) [100].

One of the potential severe complications in COVID-19 is PE [101,102]. Roncon et al. performed a systematic review including data from 7178 patients. They revealed that the incidence of acute PE among COVID-19 patients was higher in intensive care unit (ICU) patients compared to those hospitalised in general wards [103]. Other studies confirmed that PE is a significant complication of COVID-19, especially in ICU patients [104,105]. 

The rate of recurrent or progressive PE despite anticoagulant therapy is considerable. In one study, patients with confirmed PE underwent CTPA follow-up. Complete thrombus resolution was observed in 60% of the cohort. Residual thrombosis was visible in 30% of the patients [106]. In another study, complete resolution of thrombus was observed in 72% patients after a mean period of 48 days of confirmed diagnosis of PE [107]. Table 2 summarises the results of the studies mentioned above.

## 6. Conclusions

In conclusion, understanding the complex interactions between heart failure and COVID-19 is essential for optimizing patient care. Early detection and effective treatment strategies are crucial in managing these patients.

Multidisciplinary approaches, including members of the heart failure team, can help patients with chronic heart failure better understand and treat diseases, including those who receive advanced treatments during this pandemic. It is still unclear whether SARS-CoV-2 infection increased the risk of venous thromboembolism or bleeding more than respiratory infections from other diseases, such as influenza, and whether the period of thromboprophylaxis after COVID-19 should be extended. In this context, future clinical trials will be useful.

## Figures and Tables

**Table 1:** Characteristics of included studies.

	Study (Year)	Number of Patients	Design	Findings
[41]	Inciardi R.M. et al. (2020)	99	Single-centre	Out of 99 patients, 53 had cardiac disease, and 40% of them had a history of heart failure. Patients with cardiac disease had a higher mortality rate compared to those without cardiac disease (36% vs. 15%). Furthermore, patients with cardiac disease had a higher prevalence of thromboembolic incidents and septic shock compared to those without cardiac disease (23% vs. 6% and 11% vs. 0%, respectively).
[42]	Chen T et al. (2020)	274	Retrospective case series	Patients who died from COVID-19 had higher levels of troponin I, NT-proBNP, and D-dimer than those who recovered. Additionally, parameters such as alanine aminotransferase, aspartate aminotransferase, creatinine, creatine kinase, and lactate dehydrogenase were higher in deceased patients. Patients who died from COVID-19 were more likely to develop complications such as heart failure (41/83; 49%) or acute cardiac injury (72/94; 77%), and had a higher incidence of acute respiratory distress syndrome (113; 100%), type I respiratory failure (18/35; 51%), sepsis (113; 100%), alkalosis (14/35; 40%), hyperkalemia (42; 37%), acute kidney injury (28; 25%) and hypoxic encephalopathy (23; 20%). Cardiovascular complications were more common in patients with cardiovascular comorbidity. Acute cardiac injury and heart failure were more common in patients who died of COVID-19. These were independent of a history of cardiovascular disease.
[43]	Tomasoni D. et al. (2020)	692	Prospective multicentre cohort study	Patients diagnosed with heart failure were more likely to have complications such as acute heart failure (33.3% vs. 5.1%), acute renal failure (28.1% vs. 12.9%), multiorgan failure (15.9% vs. 5.8%) or sepsis (18.4% vs. 8.9%). A history of heart failure indicates a higher risk of death from COVID-19 infection (41% vs. 21%).
[44]	Alvarez-Garcia J. et al. (2020)	6439	Retrospective analysis	Patients with diagnosed heart failure were more likely to require mechanical ventilation (22.8% vs. 11.9%) and had a higher mortality rate (40.0% vs. 24.9%). Patients with previous heart failure had comparable results, regardless of the ejection fraction of left ventricle or the use of renin–angiotensin–aldosterone inhibitor.
[46]	Salah H.M. et al. (2022)	257,075	Cohort study, multicentre	Hospitalization for COVID-19 was related to increased risk of heart failure by 45%. Heart failure occurred more often in patients under 65 years of age, white, or who had been diagnosed with cardiovascular disease.
[47]	Bhatt A.S. et al. (2021)	132,312	Cohort study	Patients who were previously diagnosed with heart failure and were hospitalized due to COVID-19 had a significantly higher mortality rate (24.2%) compared to those who were hospitalized for acute heart failure (2.6%). Additionally, male gender, advanced age, morbid obesity, and diabetes were identified as risk factors associated with poorer outcomes and higher mortality during hospitalization.
[48]	Sokolski M. et al. (2021)	1282	Cohort study, multicentre, retrospective	Patients with a history of heart failure had a mortality rate of 36%, which was higher than the mortality rate of patients without a history of heart failure (23%). During hospitalization, 15% of patients experienced an acute heart failure incident, and 40% of these incidents were new cases. Patients who experienced acute heart failure during hospitalization had a higher mortality rate of 48% compared to non-heart failure patients (23%).
[49]	Greene S.J. et al. (2022)	99,052	Retrospective, cohort study	Patients with worsening heart failure with reduced ejection fraction (HFrEF) and those without HFrEF exacerbation had a higher 30-day mortality compared to patients without concomitant heart failure. Among patients diagnosed with HFrEF who tested positive for COVID-19, there was a higher risk of death within 30 days and an increased likelihood that their heart failure worsened. Additionally, patients who presented to healthcare facilities as outpatients had a higher mortality rate.
[50]	Kim H.J. et al. (2022)	212,678	Retrospective, cohort study	COVID-19 infection increases the risk of developing new-onset heart failure and exacerbating pre-existing heart failure. Patients with a history of heart failure had a poorer prognosis, a higher mortality rate (17.71% vs. 9.28%), and greater risk of developing severe complications compared to patients without heart failure. However, mechanical ventilation or admission to the intensive care unit was not required more often in patients with a history of HF. In contrast, COVID-19 infection was not found to be more frequent in patients with heart failure.
[52]	Yonas E. et al. (2021)	21,640	Analysis	Patients who have been diagnosed with heart failure and develop COVID-19 are more likely to require hospitalisation (odds ratio [OR] 2.37), experience poor outcomes (OR 2.86), and have an increased risk of death (OR 3.46).
[53]	Rey J.R. et al. (2020)	3080	Prospective cohort study	Patients diagnosed with chronic heart failure (CHF) have a higher frequency of acute heart failure (AHF) episodes (11.2%) than patients without CHF (2.1%), and N-terminal pro brain natriuretic peptide levels are elevated. Additionally, CHF is associated with higher mortality rates (48.7%) than non-HF patients (19%). Arrhythmias during hospital admission and CHF were found to be the main factors contributing to the development of AHF. Patients who develop AHF have a higher mortality rate (46.8% vs. 19.7%). Discontinuation of guideline-directed medical therapy, including beta-blockers, mineralocorticoid receptor antagonists, and angiotensin-converting enzyme inhibitors or angiotensin receptor blockers, was also associated with increased mortality rates.
[54]	Zaccone G. et al. (2021)		Analysis	In patients hospitalised for COVID-19 infection, the incidence of HF comorbidity is (4–16%), this may be due to a shared cardiometabolic risk profile and comorbidities such as hypertension, diabetes, obesity and chronic kidney disease, which increase the risk of severe course of COVID-19 and are also risk factors of HFpEF. COVID-19 infection can induce acute decompensation of HF in patients with pre-existing HFpEF and in those with subclinical diastolic dysfunction. In the acute and subacute phases of COVID-19, impaired diastole (rather than systole), pulmonary hypertension, and right ventricular dysfunction can be observed. In 78% of patients in the chronic phase of COVID-19, inflammation and myocardial fibrosis are observed.
[55]	Zuin M. et al. (2022)	1,628,424	Retrospective	Cardiovascular disease and structural heart changes are more common in patients after recovery from COVID-19. Additionally, patients who underwent COVID-19 were more likely to experience an episode of HF. The overall incidence of HF after COVID-19 infection was 0.4–2%. After 9.2 months, the frequency was 1.8–2.04. Moreover, an increased risk of HF was caused by older age and hypertension.
[56]	Gryglewska-Wawrzak K. et al. (2022)	120	Single-centre	The group of study participants with %VO2pred < 80% had a significantly higher proportion of men and a higher total body water (TBW%) compared to the control group (53% vs. 29% and 52.67% (±6.41) vs. 49.89% (±4.59), respectively). Individuals who presented with limited exercise capacity after COVID-19 infection demonstrated lower tricuspid annular plane systolic excursion (TAPSE), global peak systolic strain (GLPS), and late diastolic filling (A) velocity [21.86 mm (±4.53) vs. 24.08 mm (±3.20); 19.34% (±1.72) vs. 20.10% (±1.35)%; a median of 59.5 cm/s vs. 70.5 cm/s) compared to the control group.

**Table 2:** The characteristics of included meta-analyses.

	Study (Year)	Number of Patients	Design	Findings
[93]	Bansal A. et al. (2020)	3175	Meta-analysis	Cardiac injury in patients with a COVID-19 was associated with higher risk of mortality (risk ratio [RR]:7.79; 95% confidence interval [CI]: 4.69–13.01; I^2^ = 58%), admission to the intensive care unit (ICU) (RR: 4.06; 95% CI: 1.50–10.97; I^2^ = 61%), mechanical ventilation (RR: 5.53; 95% CI: 3.09–9.91; I^2^ = 0%), and developing coagulopathy (RR: 3.86; 95% CI: 2.81–5.32; I^2^ = 0%).
[94]	Jin S. et al. (2020)	4889	Meta-analysis	Severe patients had significantly higher D-dimer levels and prolonged prothrombin time (PT) compared with non-severe patients. Non-survivors had significantly higher D-dimer levels, prolonged PT, and decreased platelet count (PLT) compared to survivors. In total, 6.2% (95% CI: 2.6–9.9%) of COVID-19 patients were complicated by disseminated intravascular coagulation (DIC), in which the log risk ratio in non-survivors was 3.267 (95% CI: 2.191–4.342, Z ¼ 5.95, *p* < 0.05) compared with that in survivors.
[95]	Polimeni A. et al. (2021)	6439	Meta-analysis	D-dimer was significantly lower in COVID-19 patients with non-severe disease than in those with severe (standardized mean difference [SMD] −2.15 [−2.73–−1.56], I^2^ 98%, *p* < 0.0001). D-dimer in survivors was lower compared to non-survivors (SMD −2.91 [−3.87–−1.96], I^2^ 98%, *p* < 0.0001). Platelet count showed higher levels of mean PLT in non-severe patients than those observed in the severe group (SMD 0.77 [0.32–1.22], I^2^ 96%, *p* < 0.001).
[96]	Zhu J. et al. (2021)	6492	Meta-analysis	Patients with severe disease showed a significantly lower platelet count (weighted mean difference [WMD]: −16.29 × 109/L; 95% CI: −25.34–7.23) and shorter activated partial thromboplastin time (WMD: 0.81 s; 95% CI: −1.94–0.33) but higher D dimer levels (WMD: 0.44 μg/mL; 95% CI: 0.29-0.58), higher fibrinogen levels (WMD: 0.51 g/L; 95% CI: 0.33–0.69) and longer prothrombin time (PT; WMD: 0.65 s; 95% CI: 0.44–0.86). The patients who died showed significantly higher D dimer levels (WMD: 6.58 μg/mL; 95% CI: 3.59–9.57), longer PT (WMD: 1.27 s; 95% CI: 0.49–2.06) and lower platelet count (WMD: −39.73 × 109/L; 95% CI: 61.99–−17.45) than patients who survived.
[97]	Zhang A. et al. (2020)	2277	Meta-analysis	The level in severe cases was lower than in mild cases, while the levels of PT, D-Dimer and fibrinogen were higher than those in mild cases (*p* < 0.05). The PT of the ICU patients was significantly longer (*p* < 0.05) than that of the non-ICU patients. PT and D-dimer were higher in non-survivors, PLT was lower than that of survivors (*p* < 0.05).
[98]	Zhang X. et al. (2020)	3952	Meta-analysis	Patients with severe symptoms exhibited higher levels of D-dimer, PT and fibrinogen than patients with less severe symptoms (SMD 0.83, 95% CI 0.70–0.97, I^2^ 56.9%; SMD 0.39, 95% CI: 0.14–0.64, I^2^ 79.4%; and SMD 0.35, 95% CI 0.17–0.53, I^2^ 42.4%, respectively).
[99]	Agarwal G. et al. (2022)	6053	Meta-analysis	Patients with COVID-19 with venous thromboembolic events (VTE) had higher leukocyte counts and higher levels of D-dimer, C-reactive protein, and procalcitonin.
[100]	Alharbi M.G. et al. (2022)	55	Meta-analysis	Immune thrombocytopenia (ITP) secondary to COVID-19 infection was slightly more common among males (54.8%) than females.
[103]	Roncon et al. (2020)	7178	Meta-analysis	Among patients with COVID-19 hospitalized in general wards and ICU, the pooled in-hospital incidence of pulmonary embolism (PE) (or lung thrombosis) was 14.7% of cases (95% CI: 9.9–21.3%, I^2^ = 95.0%, *p* < 0.0001) and 23.4% (95% CI:16.7–31.8%, I^2^ = 88.7%, *p* < 0.0001), respectively. Segmental/sub-segmental pulmonary arteries were more frequently involved compared to main/lobar arteries (6.8% vs. 18.8%, *p* < 0.001).
[104]	Ng J. J. et al. (2020)	1182	Meta-analysis	The weighted average incidence of PE in COVID-19 patients admitted to the ICU was 11.1% (95% CI 7.7% to 15.7%, I^2^ = 78%, Cochran’s Q test *p* < 0.01).
[105]	Gong X et al. (2022)	10,367	Meta-analysis	The cumulative incidence of PE in patients with COVID-19 was 21% (95% confidence interval [95% CI]: 18–24%; *p* < 0.001), and the incidence of pulmonary embolism in ICU and non-ICU patients was 26% (95% CI: 22–31%; *p* < 0.001) and 17% (95% CI: 14–20%; *p* < 0.001), respectively.

## Data Availability

Not applicable.
